# Zyxin is involved in regulation of mechanotransduction in arteriole smooth muscle cells

**DOI:** 10.3389/fphys.2012.00472

**Published:** 2012-12-20

**Authors:** Zhe Sun, Shaoxing Huang, Zhaohui Li, Gerald A. Meininger

**Affiliations:** ^1^Dalton Cardiovascular Research Center and Department of Medical Pharmacology and Physiology, University of MissouriColumbia, MO, USA; ^2^Bayer CropScienceMorrisville, NC, USA

**Keywords:** shRNA silencing, integrin, mechanical forces, fibronectin, atomic force microscopy (AFM), microcirculation, focal adhesion, extracellular matrix proteins

## Abstract

Zyxin is a focal adhesion protein that has been implicated in the modulation of cell adhesion and motility, and is hypothesized to be a mechano-sensor in integrin-mediated responses to mechanical force. To test the functional role of zyxin in the mechanotransduction of microvascular smooth muscle cells (VSMC), we utilized atomic force microscopy (AFM) to apply localized pulling forces to VSMC through a fibronectin (FN) focal adhesion induced by a FN-coated bead on cell surface. Application of force with the AFM induced an increase of zyxin accumulation at the site of the FN-bead focal adhesion that accompanied the VSMC contractile response. Whereas, reduction of zyxin expression by using a zyxin-shRNA construct abolished the VSMC contractile response to AFM pulling forces, even though the zyxin-silenced VSMCs displayed increased adhesion to FN in both AFM adhesion assays and cell adhesion assays. The reduced zyxin expression significantly impaired cell spreading and reorganization of the actin cytoskeleton that could indicate a possible underlying reason for the loss of a contractile response to mechanical force. Consistent with these observations, in zyxin-silenced VSMC, we also observed a reduced expression of Rac1, which plays an important role in the actin reorganization in VSMC, but increased thyroid receptor-interacting proteins (TRIP6) and FAK expression, the latter being a major protein that promote cell adhesion. In conclusion, these data support an important enabling role for zyxin in VSMCs ability to mechanically respond to applied force.

## Introduction

Cell and extracellular matrix (ECM) interactions play crucial roles in multiple cellular functions, such as cell adhesion, spreading, motility, and shape (Schwartz, 1997; Zamir and Geiger, 2001). These processes are mediated by formation of protein complexes at cell-ECM adhesion sites that begin as focal contact points and mature into focal adhesions. Focal adhesions are discrete, specialized regions at which cultured cells adhere to the ECM, which are considered to be similar in structure and composition to microvascular smooth muscle cells (VSMC) adhesion points in the vessel wall known as dense plaques. These structures serve as mechanical and biochemical signaling sites at which integrin receptors cluster and bind to numerous signaling and structural proteins to establish a connection between the ECM and the actin cytoskeleton to mediate outside–in and inside–out signaling events (Jockusch et al., 1995; Craig and Johnson, 1996).

Zyxin is one focal adhesion protein with a molecular weight of 82 KDa that was first isolated from chicken embryo fibroblasts (Beckerle, 1986). Zyxin is a member of LIM domain protein family that includes lipoma-preferred partner (LPP) (Gorenne et al., 2003) and thyroid receptor-interacting proteins (TRIP6) (Yi and Beckerle, 1998), all of which have been detected at focal adhesions and cell-ECM and cell–cell junctional areas. LIM domain proteins possess two distinct motifs: a proline-rich N-terminal region also containing a nuclear export signaling sequences and a C-terminal region consisting of three LIM domains (termed by the initials of ***L****in-11*, ***I****sl-1, and* ***M****ec-3*). Through the LIM domain- and the N-terminal proline-rich domain-mediated protein–protein interactions, zyxin forms complexes with molecules such as α-actinin and Mena/VASP, which are critically involved in actin polymerization at focal adhesions as well as in stress fibers (Crawford et al., 1992; Hoffman et al., 2006).

Recently, several lines of evidence have led to the postulation that zyxin may function as a mechano-sensor. First, the observation that zyxin can shuttle between a focal adhesion site and cell nucleus (Nix and Beckerle, 1997) has raised speculation that zyxin may be one of the long sought after mechano-sensors that regulate gene transcription when prompted by mechanical stimuli. Secondly, zyxin has displayed force sensitive translocation to focal adhesions and stress fibers in fibroblasts and endothelial cells (Yoshigi et al., 2005; Hirata et al., 2008a,b; Ngu et al., 2010). Thirdly, it has been reported that when traction force at focal adhesions is reduced there is an increase in the molecular unbinding kinetics (k_off_) of zyxin (Lele et al., 2006). We have previously shown that arteriolar VSMC exhibits a contractile response when a stretching force is applied perpendicular to the cell membrane through fibronectin (FN)-induced focal adhesion sites on the cell surface (Sun et al., 2008). This supports the existence of an integrin mediated mechanotransduction pathway that can lead to VSMC contraction. In this report, we tested the hypothesis that zyxin was important for this integrin-mediated mechanotransduction pathway by knockdown of zyxin expression using a siRNA approach as well as atomic force microscopy (AFM) to measure and apply forces to single focal adhesion sites on VSMC. Our results showed that zyxin modulates the VSMC mechanotransduction by influencing cytoskeletal structure and signaling pathways.

## Materials and methods

### Plasmids and antibodies

Zyxin-shRNA construct (Clone ID: V2MM_26449) targeting rat zyxin (GenBank accession number: XM_216124; targeting sites: 1622–1640) were purchased from Open Biosystems (Huntsville, AL). Negative control shRNA pRS plasmid was from Origene Technologies (Rockville, MD). Antibodies for zyxin, LPP, TRIP6, and RhoA were from Santa Cruz Biotechnology (Santa Cruz, CA). Antibodies for integrin α5, β1, β3, ILK, Rac1, VASP, paxillin, vinculin, FAK, and c-Src were purchased from Millipore (Billerica, MA). ß-actin antibody was from Sigma (St. Louis, MO). Purified collagen type I, fibrinogen, vitronectin, and laminin were from BD Biosciences (San Jose, CA) and purified FN was purchased from Invitrogen (Carlsbad, CA).

### Cell culture and transfection

All animals were handled in accordance with the guidelines of the Animal Care and Use Committee of the University of Missouri using approved protocols. VSMC were isolated from first-order feed arterioles of rat cremaster muscles according to the protocol described by Wu et al. (1998), Cells were cultured in DMEM/F-12 (Invitorgen; Carlsbad, CA) supplemented with 10% fetal bovine serum (FBS) and used in the experiments at passages 3–10. For transfection and selection of stably transfected clones, cells were transfected using Fugene 6 reagent according to the protocol described by the manufacturer (Roche; Indianapolis, IN). The ratio of Fugene 6 reagent to plasmid DNA was maintained at 3 μl of Fugene 6 reagent to 2 μg of plasmid DNA. Briefly, 94 μl of serum-free medium was mixed with 6 μl of Fugene 6 reagent and incubated for 5 min at room temperature, and then 4 μg of plasmid DNA was added to the cocktail and incubated for 45 min. This mixture was added drop wise to cells that have been resuspended in 2 ml of fresh regular culture medium at the density of 10^5^/ml, mixed well and returned to the incubator. Zyxin-GFP expression can usually be observed in 48 h. To establish stably transfected VSMC clones, 6 days after the start of transfection, mixed culture of stably transfected VSMC clones were obtained by treating the transfected cells with 2.5 μg/ml puromycin (Invitrogen, Carlsbad, CA) continuously for 6–8 weeks.

### Cell-adhesion assay

Adhesion assays were carried out in 96-well plates pretreated with different substrates. The plates were washed with PBS twice and then blocked with 0.1% BSA in regular culture medium at 37°C for 1 h. After cells were trypsinized and resuspended in serum free DMEM-F12 medium supplemented with 0.1% BSA, 100 μl of resuspended cells (2 × 10^5^/ml) was placed to each well and returned to 37°C incubator for 1 h. Non-adherent cells were washed off with PBS twice and attached cells were fixed with 2% paraformaldehyde at room temperature for 15 min. Attached cells were washed and stained with Crystal Violet (1 mg/ml in purified water) for 10 min at room temperature. The plates were allowed to dry completely and 50 μl of 2% SDS was placed to each well after being washed with water. After incubating at room temperature for 30 min, the plates were read at 560 nm on a Bio-Tek Synergy HT plate reader.

### Cell spreading assay

Cell spreading assays were performed by plating cells on 50 μg/ml FN-coated dishes for 1, 3, and 5 h under standard culture conditions. Cells were then fixed and stained with Alexa 568 conjugated-phalloidin. Images were taken with an Olympus confocal microscope. Cell areas were determined using NIH Image J software.

### Cell migration assay

The migration assays were undertaken following protocols described by Chao et al. (2006). Briefly, A Boyden chamber with 48 wells and membranes with pores of 8 μm (Neuro Probe, Gaithersburg, MD) were used. The membranes were pretreated with 50 μg/ml FN overnight at 4°C and blocked with DMEM/F-12 medium supplemented with 0.1% BSA. In each well of the lower chamber, 30 μl of DMEM/F-12 containing 0.1% BSA and 50 μg/ml FN were placed as the chemoattractants, and in each well of the upper chamber, 50 μl of cells (4 × 105/ml) was added. After incubation at 37°C for 3 h, cells in the upper chamber and non-migrated cells on the topside of the membrane were removed, and migrated cells on the lower side of the membrane were fixed and stained with Diff-Quik stain kit following instruction by the manufacturer (Fisher Scientific, Pittsburg, PA). The membranes were then scanned and staining intensity of migrated cells was determined with a densitometric method (NIH Image J).

### Atomic force microscopy

The method previously described by Sun et al. (2005, 2008) was used for mechanical force measurement. Briefly, an atomic force microscope (Bioscope; model IVa; Digital Instruments, Santa Barbara, CA) mounted on an inverted microscope (Olympus, IX81, Long Island, NY) was used in contact mode operation. To measure the VSMC mechano-responsiveness to axial pulling forces, silicon nitride cantilevers (model MLCT-AUHW; Veeco, Camarillo, CA) with a spring constant of 0.01 N/m were used (Novascan, Ames, IA). Cantilevers were tipped with a 5-μm bead coated with PEG-biotin and FN. The beads were incubated with 3 mg/ml avitin (Sigma, St. Louis, MO) then washed with DPBS and labeled with 1 mg/ml FN-biotin conjugates, followed by washing with DPBS to remove the unbound FN. The cantilever tips with FN-coupled beads were put onto the AFM and brought into contact with the cell surface and maintained for 20 min, during which period FN on the bead interact with integrin receptors. Mechanical forces were then applied by pulling the cantilever tip upward in the axial direction of the z-axis, and the subsequent bead movement was recorded using AFM, which reflected the generation of a VSMC mechanical response to the applied pulling force. Experiments were carried out in DMEM/F-12 medium at room temperature. Matlab software (Math-Works, Natick, MA) was used to analyze the VSMC mechanical response to forces. To further quantify and compare the cellular mechanical responses, data were analyzed and the cell responses expressed as fractional compensation (*f*_*c*_) according to the formula:

$$f_{c}=\left(D_{\text{AFM}}-D_{\text{cell}}\right)/D_{\text{AFM}}$$

**Figure 1:**
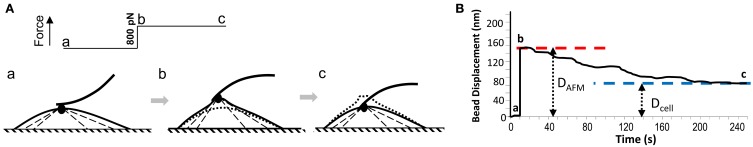
**(A)** A schematic illustration of AFM pulling experiment on VSMC cell: a, AFM cantilever probe with a FN-coated bead at the tip forms focal adhesion on VSMC surface; b, AFM pulling force (800 pN) was applied to FN-focal adhesion by altering the cantilever deflection, and lifted the FN-bead and focal adhesion; c, while AFM pulling force was maintained constant, VSMC developed contraction and pulled the FN-bead and focal adhesion downward. **(B)** AFM recording of the bead displacement during the pulling experiment shown in **(A)**. D_AFM_ and Dcell were measured as shown and were used to calculate the cell compensation.

*D*_AFM_: the initial distance that bead was lifted in the z-axis following the pull with the AFM cantilever (Figures 1A,B).

*D*_cell_: the ending distance that the bead was from its pre-pulling position after the cell's local contractile response (Figures 1A,B).

For the adhesion probability and rupture force measurement, AFM probes were washed with water and incubated with PEG (Molecular weight: 8000, 10 mg/ml) for 10 min at room temperature. After washing with water, the probes were coated with FN (1 mg/ml) for 10 min. After washing, the AFM probe was interacted with the cell surface in a cyclic fashion to approach, contact and then retract from the cell membrane in force mode operation. During AFM probe retraction, if a specific binding event occurred, the rupture of this binding (failure force) was detected as a small sharp shift in the retraction curve reflecting an abrupt change in cantilever deflection. The rupture forces of integrin-ECM interactions were analyzed using NforceR software (Trzeciakowski and Meininger, 2005). The sampling order was assigned randomly. For each of the control and zyxin-shRNA groups, 1000 force curves were pooled from 20 randomly selected cells (50 curves per cell) with each probe. The adhesion probabilities were determined as the number of force curves with binding events divided by the number of total force curves pooled.

### Confocal laser scanning microscopy

Cells at 50–70% confluency were trypsinized and plated on 35 mm culture dishes (FluoroDish, World Precision Instruments; Sarasota, FL). Cells were incubated with primary antibodies (1:200 dilution-μ g/ml) in antibody buffer (15 mM Na_3_C_6_H_5_O_7_, 150 mM NaCl, 2% BSA, and 0.05% Triton X-100) at 4°C overnight after being fixed with 2% paraformaldehyde and washed with 0.1 mM glycine buffer. Cells were incubated with Alexa 488-conjugated secondary antibody (goat anti-rabbit, goat anti-mouse, or rabbit anti-goat IgG) (1:100 dilution-μg/ml) and/or Alexa 568-conjugated phalloidin (1:100-μg/ml dilution) in a dark environment for 1 h after being washed with cold buffer (15 mM Na_3_C_6_H_5_O_7_, 150 mM NaCl, and 0.05% Triton X-100). After cells were washed with cold buffer, images of the cells were taken on a confocal microscope (Olympus FV 1000) using excitation wavelengths of 488 nm and 561 nm, respectively. Image analysis was performed using the Olympus FV 1000 software.

### Western blot analysis

After being washed twice with ice-cold PBS, cells were lysed in CSK buffer (50 mM NaCl, 300 mM sucrose, 3 mM MgCl_2_, and 10 mM PIPES at pH6.8) supplemented with 1 μg/ml pepstatin and a complete protease inhibitor cocktail (Roche, Indianapolis, IN). The BCA Protein Assay Kit (PIERCE; Rockford, IL) was used to measure protein concentrations. Equal amounts of total proteins were loaded on SDS-PAGE gels and transferred to PVDF membranes using the NuPAGE electrophoresis system from Invitrogen (Carlsbad, CA). After being incubated in blocking buffer (TBS containing 2% milk and 0.1% Tween 20) for 30 min at room temperature, the membrane was incubated in blocking buffer with primary antibodies at 4°C overnight. After being washed with TBS containing 0.1% Tween 20 for 6 times, the membrane was visualized using the Visualizer Western Blot Detection Kit (Millipore, Billerica, MA). For loading control, the membranes were stripped using Stripping Buffer II (Upstate; Lake Placid, New York) and incubated with anti-ß-actin antibody. The protein bands on the films were quantified using DNR's GelQuant software (DNR Bio-Imaging Systems Ltd, Jerusalem, Israel) and normalized to the corresponding ß-actin band.

### Statistical analysis

Experimental data are presented as means ± SEM. Each experiment was repeated at least three times and representative results are shown. Statistical analyses were performed using student *t*-test on Microsoft Excel and ANOVA analysis of SAS9.1. *P* < 0.05 was considered statistically significant.

## Results

### Force-induced recruitment of zyxin-GFP to FN focal adhesion sites

**Figure 2:**
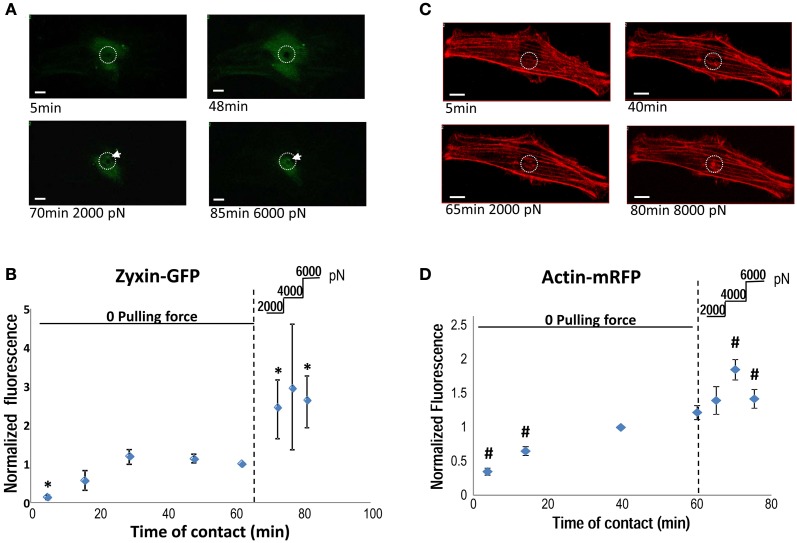
**(A)** Accumulation of zyxin-GFP around a FN-coated microsphere (10 μm) applied on the VSMC surface using AFM. FN-bead AFM probes were engaged to cell surface for 65 min before pulling forces were applied stepwise (2000 pN/step). The white circle illustrate the position of FN-bead, and white arrow pointing to the zyxin clustering. **(B)** The time course of zyxin-GFP recruitment to the FN-bead adhesion site quantified as normalized fluorescence intensity. **(C)** Accumulation of actin-RFP around a FN-coated microsphere (10 μm) applied on the VSMC surface using AFM. The white circle illustrate the position of FN-bead. **(D)** The time course of actin-RFP recruitment to the FN-bead adhesion site quantified as normalized fluorescence intensity. A z-stack of through-focus confocal fluorescence images were captured through the whole cell depth at each time point. Images containing signals corresponding to the surface of FN-bead were stacked together, quantified and normalized to the sum of zyxin (actin)-recruitment at *t* = 65 (60 min). *N* = 5 experiments, respectively; data presented as mean ± SEM. ^*^p < 0.05 compared to zyxin accumulation at *t* = 65 min; ^#^*p* < 0.05 compared to acin accumulation at *t* = 60 min. Scale bar = 10 μm.

To visualize the participation of zyxin in the VSMC mechanotransduction, VSMCs were transiently transfected with a zyxin-GFP construct. A FN-coated microsphere immobilized at the tip of an AFM probe was applied to cell surface to induce focal adhesion, and to mediate the AFM pulling forces. As shown in Figures 2A,B, AFM pulling force significantly augmented the accumulation of zyxin-GFP to the FN-adhesion site indicating that the zyxin translocation was force sensitive in VSMC. Since zyxin has been shown to regulate actin polymerization at focal adhesions, the changes of actin reorganization after the application of pulling force were also tested using similar approach and an actin-mRFP construct. As shown in Figures 2C,D, the actin reorganization also occurred at the pulling site in minutes after the force was applied through FN-coated bead, a time scale very similar to that of zyxin.

### Suppression of zyxin expression by shRNA silencing

**Figure 3:**
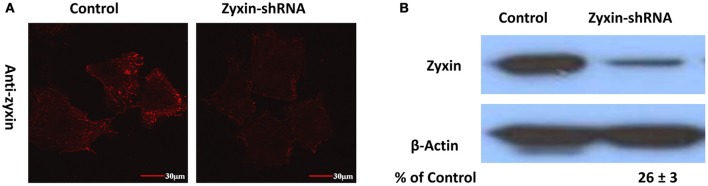
**Immunofluorescence (A) and Western blots (B) probed with anti-zyxin antibody showed that zyxin expression was reduced in VSMC stably expressing zyxin-shRNA construct in comparison with cells expressing non-silencing control.** Images were taken with a 60x oil objective. Scale bars, 30 μm. Blots were quantified and normalized to β-actin and displayed as percent of control. Control and Zyxin-silenced samples were run on the same immunoblot and were from the same film exposure. The results are representative of three independent experiments.

To reduce zyxin expression levels in VSMC, cells were transfected with a zyxin-shRNA construct and stably expressed clones were obtained by treating the transfected cells with 2.5 μg/ml puromycin continuously. A separate group of cells stably expressing a negative non-silenced construct were used as control. As shown by immunofluorescent labeling probed with an anti-zyxin antibody, zyxin expression level was significantly reduced in VSMC stably expressing zyxin-shRNA construct in comparison with control cells (Figure 3A). Western blot analysis confirmed that there was a significant reduction in zyxin protein expression level (26 ± 3% of control) in zyxin-shRNA silenced cells compared to non-silenced control cells (Figure 3B).

### Zyxin shRNA silencing affected VSMC mechanical response to pulling forces

**Figure 4:**
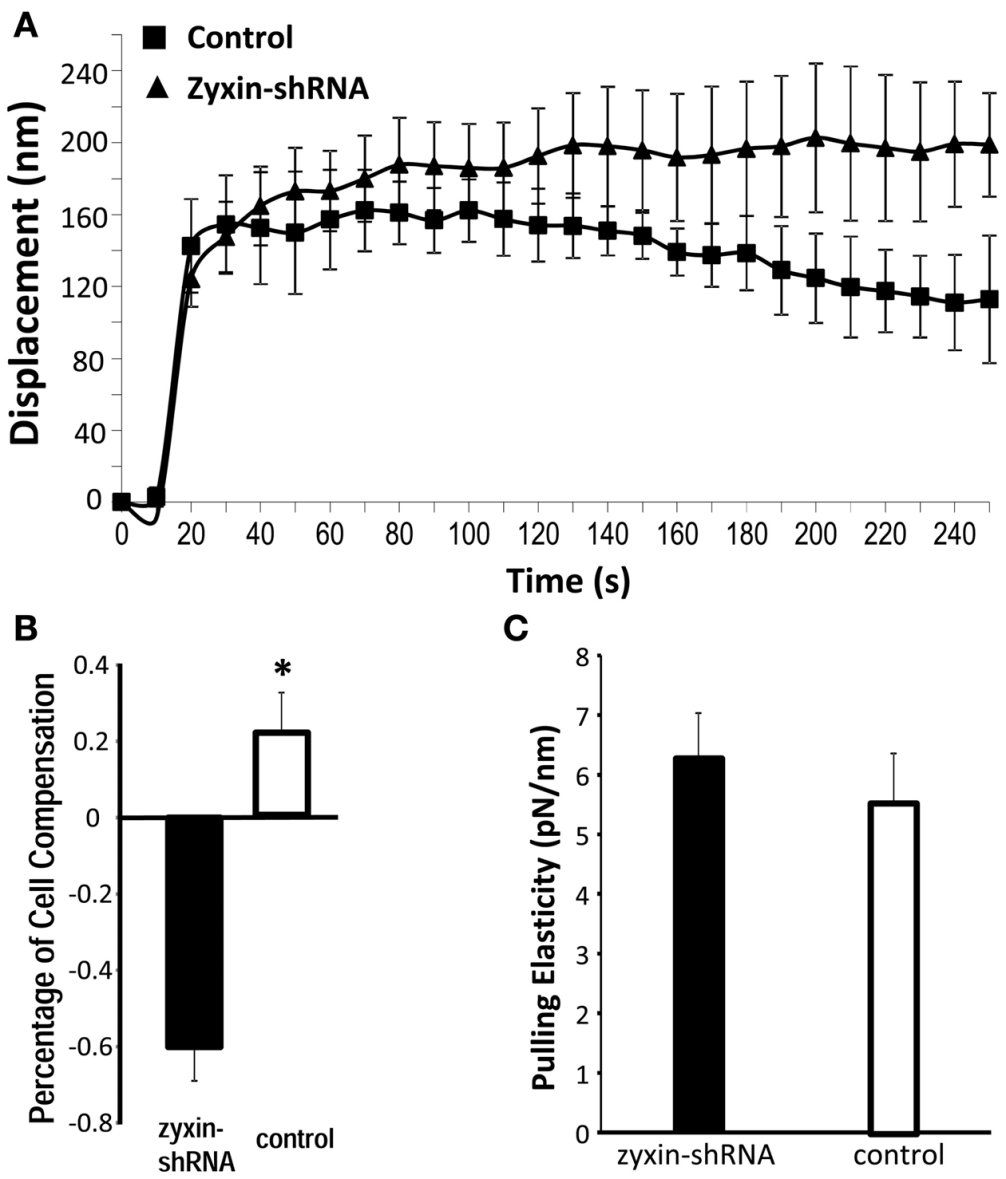
**Zyxin silencing reduced pulling force response in VSMC.** The AFM probe with FN-coated beads were placed into contact with the cell surface of VSMC (zyxin-shRNA or control). AFM pulling forces were then applied to the bead from the cell surface in z-axis. The displacement (nm) of the bead over 250 s was shown to indicate the VSMC response to the pulling force. (*n* = 5, *p* < 0.004 vs. control). **(B)** Percentage of cell compensation as an indicator of quantified cellular micro-myogenic response. **(C)** Comparison of the apparent pulling elasticity indicating no significant change in the FN-focal adhesion strength. Error bars represent standard error of the mean. ^*^p < 0.05.

Similar to previous studies (Sun et al., 2008) from our group VSMCs developed contractile responses to a pulling force (800 pN) applied with the AFM to a single FN-integrin adhesion (Figure 4A). After zyxin silencing the VSMC's response to mechanical pulling force was significantly diminished (Figure 4A). As shown in Figure 4B, the zyxin-silenced VSMC failed to generate a positive compensatory mechanical response (−60% vs. 22.5% for control). There was no significant difference in the tensile elasticity (Figure 4C), indicating that zyxin-silencing did not affect the strength of focal adhesions in VSMC.

### Zyxin shRNA silencing enhanced cell adhesion

**Figure 5:**
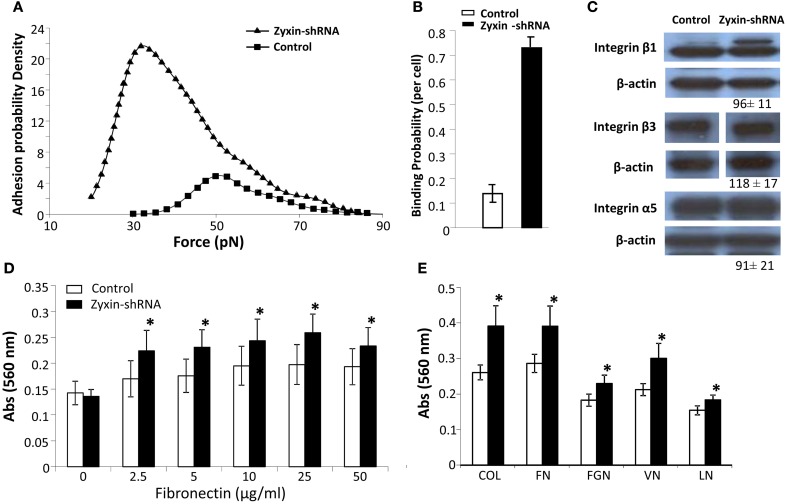
**Zyxin silencing enhanced VSMC adhesion. (A)** Distribution of integrin-FN adhesion force (unbinding or rupture forces) measured using atomic force microscopy adhesion assay. **(B)** Zyxin-shRNA cells exhibited an increased binding probability to fibronectin. The binding probability was calculated as: number of force curves with adhesions/the total number of force curves recorded. **(C)** The expression level of a5, b1, and b3 integrins was not altered by zyxin silencing as compared to control VSMC. Control and Zyxin-silenced samples were run on the same immunoblot and were from the same film exposure. **(D,E)** Zyxin silencing enhanced VSMC cell adhesion as measured with cell adhesion assay. VSMC stably transfected with control or zyxin-shRNA construct were plated with serum free culture medium in 96-well plates coated with **(D)** fibronectin at concentrations of 0, 2.5, 5, 10, 25, and 50 μg/ml or **(E)** with different substrates, and were allowed to adhere for 1 h under standard culture conditions. COL, collagen 10 mg/ml; FN, fibronectin 25 mg/ml; FGN, fibrinogen 25 mg/ml; VN, vitronectin 10 mg/ml; LN, laminin 10 mg/ml. Results represent mean ± SE of five independent experiments. (*p < 0.05 vs. control).

It has been suggested that zyxin may play a role in cell signaling in the control of cell adhesion, focal adhesion assembly, and actin stress fiber remodeling (Beckerle, 1986; Crawford et al., 1992; Golsteyn et al., 1997). In order to understand the underlying mechanisms of how zyxin silencing was reducing mechano-responsiveness in VSMC, cell adhesion of Zyxin-silenced cells were measured using both AFM adhesion assay and whole cell adhesion assays. Interestingly, Zyxin-shRNA silencing cells displayed enhanced binding to FN compared to non-silenced control cells (*n* = 20, *p* < 0.001) as shown in Figures 5A,B. In addition, there was a left-ward shift in the distribution of unbinding forces to lower levels that was observed in the Zyxin-shRNA silencing cells (Figure 5A). There was no significant difference in the protein expression levels of integrins β1, β3, and α5 between VSMCs expressing zyxin-shRNA construct and non-silencing control construct (Figure 5C). Further tests were also performed using 96-well plate adhesion assays, and the results further confirmed that silencing of zyxin by shRNA enhanced cell adhesion significantly not only on FN (Figure 5D), but also on collagen, fibrinogen, vitronectin and laminin (Figure 5E).

### Zyxin shRNA silencing impaired VSMC spreading and actin remodeling

**Figure 6:**
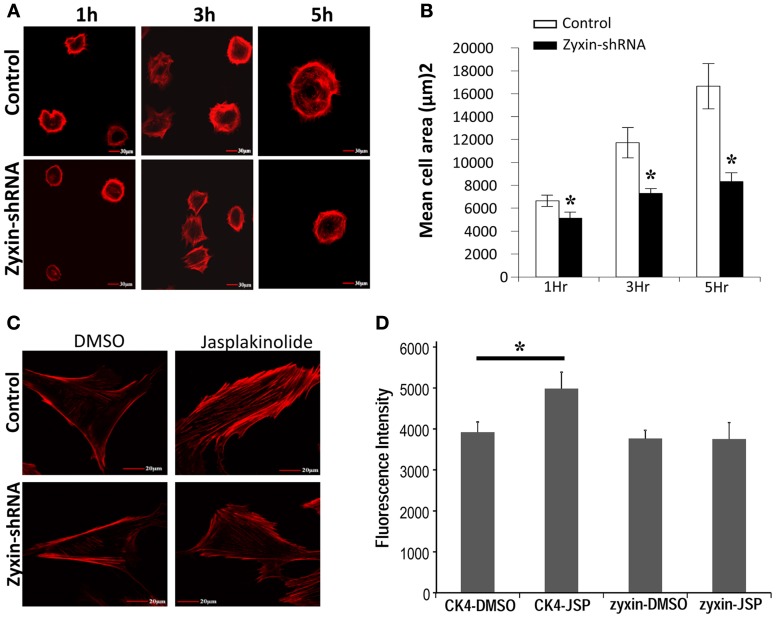
**Zyxin silencing inhibited VSMC actin cytoskeleton reorganization. (A)** silencing of zyxin inhibited VSMC spreading. Non-silencing control or zyxin-shRNA transfected VSMCs were suspended with serum-free culture medium and plated on 50 μg/ml fibronectin-coated dishes for 1, 3, and 5 h under standard culture conditions. Cells were then fixed and stained with Alexa 568 conjugated-Phalloidin. Images were taken with a 40x oil objective. Scale Bars, 30 μm. **(B)** Bar graphs represent mean of cell spreading areas calculated using NIH Image J (^*^p < 0.05 vs. control). Error bars represent error of the mean. Results are representative of five independent experiments. **(C)** Silencing of zyxin inhibited jasplakinolide-induced actin-filament strengthening. **(D)** Bar graphs represent mean of actin-staining fluorescence intensity of individual cells. CK4-DMSO, *n* = 15; CK4-JSP, *n* = 13; zyxin-DMSO, *n* = 13; and zyxin-JSP, *n* = 13. VSMCs stably transfected with non-silenced control or zyxin-shRNA were plated with regular culture medium on 50 μg/ml fibronectin-coated dishes overnight and cells were treated with 100 nM Jasplakinolide for 1 h under standard culture conditions. Cells were then fixed and stained with phalloidin. The fluorescence intensity of individual cells was calculated as the sum of a z-stack trans-focus images that were captured using confocal microscopy. Scale bars, 20 μm. (^*^p < 0.05 vs. control). Results are representative of three independent experiments.

Zyxin has been shown to translocate to stress fibers, and has been indicated to interact with VASP and actinin in promoting the actin polymerization (Crawford et al., 1992; Hoffman et al., 2006). Two experiments were performed to examine whether reorganization of actin filaments was affected in zyxin-silenced VSMC. First, the cell spreading process was compared between zyxin-silenced VSMC and control VSMC. It is known that the process of cell spreading after the initial adhesion depends on a continuous actin reorganization process. As shown in Figures 6A,B, the progress of cell spreading was significantly slowed in zyxin-silenced VSMC compared to control, suggesting that zyxin silencing weakened the actin filament remodeling in VSMC. A second method to access the actin filament reorganization is to examine actin filaments polymerization after treating the cells with jasplakinolide, which is a membrane-permeable agent that reduces the critical concentration of actin monomers required for actin polymerization (Bubb et al., 2000). Non-silenced control cells treated with jasplakinolide exhibited significantly enhanced actin stress fibers as observed by phalloidin staining, whereas zyxin-shRNA cells treated with jasplakinolide for an equal period of time exhibited fewer actin stress fibers (Figures 6C,D), further suggesting that zyxin silencing attenuated the actin polymerization.

### Changes of other focal adhesion proteins in zyxin-silenced VSMC

**Figure 7:**
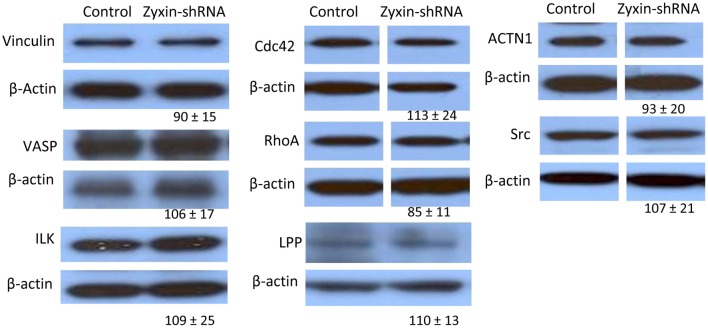
**Western blot results showing that zyxin silencing did not significantly affect the expression of a number of focal adhesion proteins: Vinculin, VASP, Actinin 1,Cdc42, c-Src, RhoA, ILK, and LPP, as compared between cells stably expressing zyxin-shRNA and cells expressing non-silencing control.** Control and Zyxin-silenced samples were run on the same immunoblot and were from the same film exposure. The results are representative of three independent experiments.

**Figure 8:**
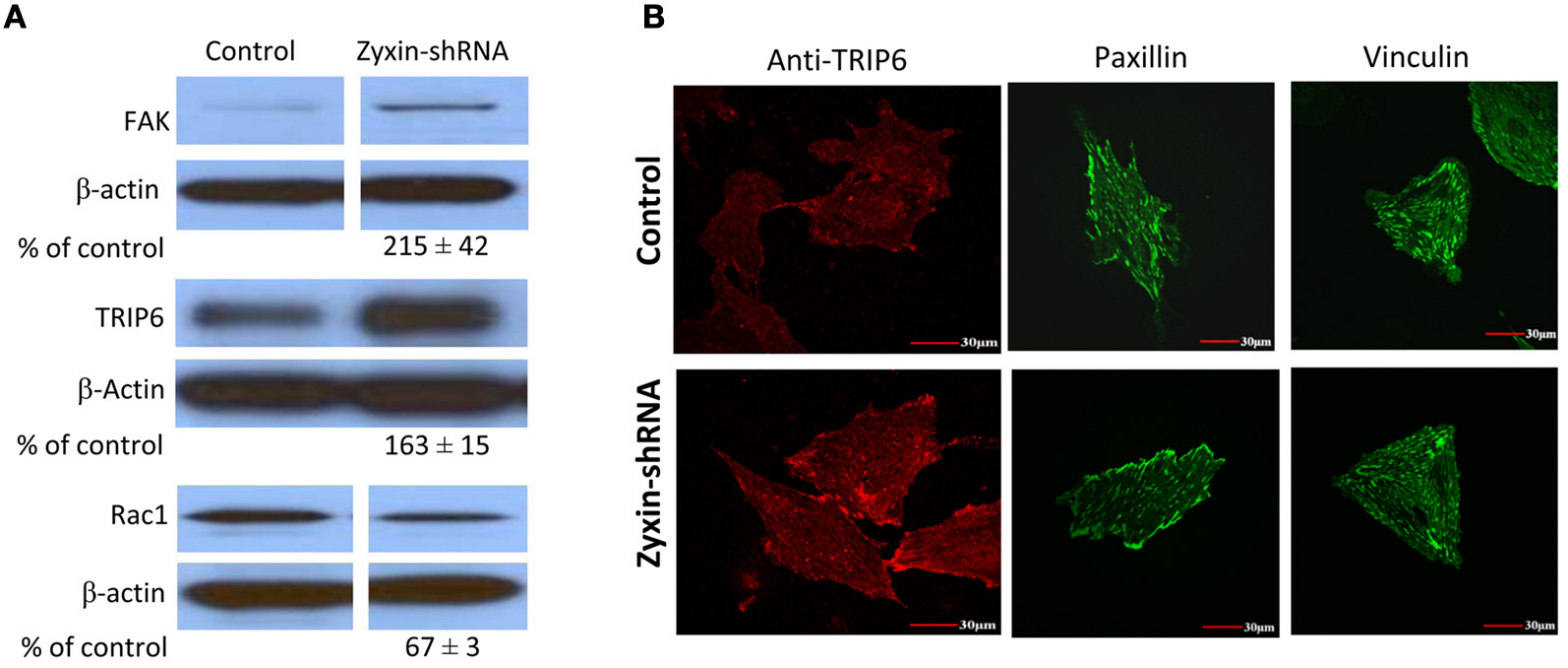
**Zyxin silencing increased FAK and TRIP6 expression, but decreased Rac1 expression in VSMC. (A)** Western blots showed that FAK and TRIP6 expression were increased, but Rac1 was decreased in cells stably expressing zyxin-shRNA compared to cells expressing non-silencing control. Blots were quantified and normalized to β-actin and presented as percentage of the control. **(B)** Immunofluorescence microscopy showed increased presence of TRIP6 in the focal adhesions of VSMCs expressing zyxin-shRNA, but no significant change was observed in the focal adhesion presence of paxillin and vinculin. Control and Zyxin-silenced samples were run on the same immunoblot and were from the same film exposure. The results are representative of three independent experiments. Scale bars, 30 μm.

Zyxin possesses a nuclear export sequence at its N-terminal region and has also been shown to translocate to the nucleus during cell stretching (Nix and Beckerle, 1997; Ngu et al., 2010), suggesting that zyxin may possess functions other than modulating focal adhesions and actin cytoskeleton in cells. Therefore, besides the absence of zyxin in the focal adhesions, there is possibility that zyxin-silencing can affect other cellular processes that may contribute to the impaired VSMC mechano-response to pulling force. A set of western blot experiments were performed to determine whether there were changes in level of other focal adhesion proteins in zyxin-silenced VSMC. Among the proteins tested, there were no changes in the expression levels of vinculin, VSAP, cdc42, Src, RhoA, actinin 1, ILK, and LPP (Figure 7). However, significant increases of focal adhesion kinase (FAK, 215 ± 42% of control, *n* = 5, *p* < 0.05) and thyroid receptor-interacting protein 6 (TRIP6, 163 ± 15% of control, *n* = 4, *p* < 0.01) expressions were observed in zxyin-silenced VSMC compared to control. In addition, a significant decrease of Rac1 expression (67 ± 3% of control, *n* = 3, *p* < 0.01) was also evident (Figure 8A). Immunofluorescence labeling also demonstrated there were no changes in the presence of paxillin, vinculin in focal adhesions, while a significantly stronger presence of TRIP6 at focal adhesions was observed in zyxin-silenced VSMC (Figure 8B).

## Discussion

Zyxin is a phosphoprotein that has been implicated in modulation of multiple cellular functions, including cell growth, cell motility, cell adhesion, stress fiber formation, and tumor growth (Golsteyn et al., 1997; Hoffman et al., 2006; Sy et al., 2006). We showed here that AFM pulling force-induced accumulation of zyxin to FN-focal adhesions in VSMC, whereas knock-down of zyxin expression by transfecting VSMC with a zyxin-shRNA construct abolished cell contraction in response to AFM pulling force applied through FN-focal adhesions. This evidence supports zyxin involvement in the integrin-mediated mechanotransduction in VSMC. Further experiments revealed that RNAi-silencing of zyxin did not alter integrin expression but significantly enhanced cell adhesion with ECM proteins (FN, CN type I, Laminin, vitronectin, and fibrinogen). Reducing zyxin expression also slowed cell spreading and the process of actin reorganization in VSMC. The latter effect could explain the abolishment of VSMC mechano-response. In addition, there was also a significant increase of TRIP6 and FAK expression, but a decrease of Rac expression in zyxin-silenced VSMC compared to control. These results provide new insights into the multi-functional role of zyxin in modulating the VSMC mechanotransduction.

Mammalian cell focal adhesions have been an area of focused research for the past two decades. Much of this work has aimed at understanding the mechanism of cellular mechano-transduction (Naruse et al., 1998; Chen et al., 1999; Bershadsky et al., 2003; Helmke et al., 2003), and also raised interests that focal adhesions could serve as a mechanosensor in VSMC both *in vivo* and *in vitro* (Intengan and Schiffrin, 2000; Schwartz et al., 2003). Zyxin belongs to a small group of focal adhesion molecules, which display translocation when cells were exposed to mechanical force. Zyxin has been shown to translocate from focal adhesions to stress fibers and the nucleus in cyclically stretched fibroblasts and endothelial cells (Yoshigi et al., 2005; Ngu et al., 2010). Furthermore, release of cytoskeletal stress in fibroblast caused zyxin to dissipate from focal adhesions into the cytosol (Rottner et al., 2001; Hirata et al., 2008a), while application of external forces recruited zyxin from cytosol into focal adhesions (Hirata et al., 2008a). Collectively, evidence points to zyxin as a protein involved in cellular processes that are mechano-sensitive. In this report, our results confirmed that zyxin was recruited to FN focal adhesions that were subjected to locally applied mechanical forces. Interestingly, the recruitment of actin cytoskeleton underneath the FN-adhesion site was also enhanced by pulling forces following a similar time scale (Figure 2). These observations support a role for zyxin in modulating cytoskeletal and signaling behavior during mechanotransduction in VSMC.

The N-terminal proline-rich domain of zyxin has been shown to form complexes with Mena/VASP family proteins in facilitating actin-polymerization at focal adhesions and stress fibers in fibroblast (Yoshigi et al., 2005; Hoffman et al., 2006). Consistent with these observations, our data demonstrated that zyxin silencing impaired the processes of actin polymerization and reorganization in VSMC (Figure 4). Previous studies have shown that actin polymerization is critically involved in the localized VSMC contractile response to FN-mediated mechanical forces. For example, the VSMC contractile mechano-response to pull at an FN focal adhesion was totally abolished by Cytochalasin-D, but was enhanced in the presence of jasplakinolide (Sun et al., 2008). Therefore, we considered that the impaired actin-polymerization in zyxin-silenced VSMC provided a possible explanation of the abolished force-induced contractile response. Interestingly, in Zyxin-silenced VSMC, we also observed a significant down-regulation of Rac1 expression (Figure 6A). Rac1 is member of small GTPase family protein, and is central to the regulation of actin-polymerization that drives the lamellipodia in fibroblast and VSMC (Lamarche et al., 1996; Pelletier et al., 2005). It is plausible that down-regulation of Rac could also contribute to the down-regulation of actin-polymerization at the FN-adhesion site. Collectively, these data suggested that zyxin could be an important modulator promoting the actin-polymerization in VSMC by directly complexing with Mena/VASP, while a possible correlation between zyxin and Rac1 expression is worth further investigation.

Hoffman et al. has shown that the zyxin-null fibroblasts exhibited augmentation in cell adhesion and motility (Hoffman et al., 2006). In agreement with these data, in the zyxin-silenced VSMC, we also observed enhanced cell adhesion (Figure 3) and migration (data not shown). The enhanced VSMC adhesion was not accompanied by an increase of integrins that typically bind FN (Figure 5C), but could be associated with the increased expression level of FAK (Figure 6A). The increase of FAK expression has been observed in a broad range of carcinomas, and was generally associated with increased integrin-ECM adhesion and cell motility (Gabarra-Niecko et al., 2003). It is plausible that the increase of VSMC adhesion is mediated by the increased FAK activity. Unlike FAK, there was no evidence in the literature suggesting that zyxin or the up-regulated TRIP6 are directly involved in the regulation of integrin adhesion. TRIP6, another member of the zyxin family LIM domain proteins, (Yi and Beckerle, 1998; Lin and Lin, 2011) can also be targeted to focal adhesions. TRIP6 binding partners include supervillin and lysophosphatidic acid (LPA) 2 receptor (Xu et al., 2004; Takizawa et al., 2006), and has been suggested to facilitate focal adhesion turnover in epithelial cells and to mediate LPA-induced cell migration (Xu et al., 2004; Takizawa et al., 2006). Similar to zyxin, RNAi mediated knock-down of TRIP6-induced increased cell adhesion to FN in a fibroblast-like cell line COS7 (Takizawa et al., 2006), implicating that TRIP6 may not contribute to the increased VSMC adhesion.

In addition to enhanced cell-ECM adhesion in zyxin-silenced VSMC, we also observed a left shift of the integrin-FN adhesion force using AFM adhesion analysis, suggesting a possibility that zyxin, FAK or TRIP6 could be regulating the binding activity of integrin with ECM proteins. This observation is consistent with data reported by Ngu et al. that silencing of zyxin enhanced cell adhesion but reduced the initial adhesion strength in endothelial cells (Ngu et al., 2010). It is interesting that the improved VSMC adhesion, by itself, did not enhance the mechano-responsiveness of the VSMC. The reason for this is not clear. It may suggest that the ability to develop a contractile response depends more on unique compositional and structural characteristics of the focal adhesion complex underlying the adhesion site. As an example, the down-regulation of zyxin and Rac1 seem to be sufficient to prevent development of a mechanically responsive focal adhesion site with FN.

Zyxin possesses an N-terminal nuclear transport signal and has been postulated to shuttle between focal adhesion sites and the cell nucleus as a way of mediating protein expression changes that accompany cellular mechanotransduction (Nix and Beckerle, 1997; Hervy et al., 2006). In this regard, Cattaruzza et al. have reported that silencing of Zyxin altered mechanical stress-induced gene expression in VSMC (Cattaruzza et al., 2004). In this study, altered expression of FAK, TRIP6, and Rac were observed in zyxin-silenced VSMC compared to control. Interestingly, a decreased expression of zyxin has been reported in laryngeal squamous cell carcinoma (Wu et al., 2011), while an increase of FAK expression has also been reported in the head and neck squamous cell carcinomas (Agochiya et al., 1999), supporting a possible correlation between FAK and zyxin expression levels. It should be noted that, while changes of these protein levels were found in zyxin-silenced cells, how the gene expression of FAK, TRIP6, and Rac1 was regulated and whether zyxin is directly involved in the regulation of these proteins is not clear. Future investigation will be needed to determine the possible interactions/crosstalks between zyxin and FAK, TRIP6 and Rac1.

In summary, the results reported here demonstrate that zyxin plays an important role in mechanotransduction of microvascular VSMC and the ability of VSMC to respond to mechanical force with a contractile change. A relationship between zyxin and functions involving the actin cytoskeleton likely enable it's role in modulating mechanosensitivity focal adhesion sites in VSMC.

### Conflict of interest statement

The authors declare that the research was conducted in the absence of any commercial or financial relationships that could be construed as a potential conflict of interest.
